# Structural Characterization of the Degradation Products of a Minor Natural Sweet Diterpene Glycoside Rebaudioside M under Acidic Conditions

**DOI:** 10.3390/ijms15011014

**Published:** 2014-01-14

**Authors:** Indra Prakash, Venkata Sai Prakash Chaturvedula, Avetik Markosyan

**Affiliations:** 1 Organic Chemistry Department, The Coca-Cola Company, Global Research and Development, One Coca-Cola Plaza, Atlanta, GA 30313, USA; E-Mail: saipc@bluecal-ingredients.com; 2 Pure Circle SDN BHD, Lengkuk Teknologi, 71760 Bandar ENSTEK, Negeri Sembilan, Malaysia; E-Mail: avetik@purecircle.com

**Keywords:** rebaudioside M, stability, pH, temperature, degradation products, spectroscopic data, structure characterization

## Abstract

Degradation of rebaudioside M, a minor sweet component of *Stevia rebaudiana* Bertoni, under conditions that simulated extreme pH and temperature conditions has been studied. Thus, rebaudioside M was treated with 0.1 M phosphoric acid solution (pH 2.0) and 80 °C temperature for 24 h. Experimental results indicated that rebaudioside M under low pH and higher temperature yielded three minor degradation compounds, whose structural characterization was performed on the basis of 1D (^1^H-, ^13^C-) & 2D (COSY, HSQC, HMBC) NMR, HRMS, MS/MS spectral data as well as enzymatic and acid hydrolysis studies.

## 1. Introduction

Recently, many soft drink manufacturers have driven their focus towards naturally occurring high-potency sweeteners to reduce calories by introduction of non-caloric sweeteners into their beverage systems. *Stevia rebaudiana* (Bertoni), a perennial shrub of the Asteraceae (Compositae) family native to certain regions of South America (Paraguay and Brazil) [1,2] is one such example in recent years which resulted in the isolation of several potently sweet diterpenoid glycosides namely rebaudiosides A and D, stevioside, and dulcoside A; also known as stevia sweeteners of which stevioside and rebaudioside-A are the major compounds. These compounds are all glycosides of the diterpene *ent*-13-hydroxykaur-16-en-19-oic acid known as steviol [3,4]. Recently we have reported the isolation and sensory evaluation of rebaudioside M (**1**, also known as rebaudioside X), a minor constituent from *S. rebaudiana* Bertoni, which is about 160–500 times sweeter than sucrose [5]. Due to continuing demand of minor steviol glycosides like rebaudioside D and M, there has been intense interest on *S. rebaudiana* and that is why it is grown commercially in a number of countries, particularly in Japan, Taiwan, Korea, Thailand, Indonesia and China.

As a part of our continuing research to discover natural sweeteners, we have recently isolated several novel diterpene glycosides from the commercial extracts of the leaves of *S. rebaudiana* obtained from various suppliers around the world [6–9]. Apart from isolating novel compounds from *S. rebaudiana* and utilizing them as possible natural sweeteners or sweetness enhancers, we are also engaged in synthesis of various novel steviol glycosides and understanding their physicochemical profiles as well as their stability in various systems of interest [10–14]. We are also engaged in studying the stability data of various major steviol glycosides like rebaudioside A and stevioside under fluorescent and acidic conditions by isolating and characterizing their degradation products using various spectroscopic and chemical studies [15–17]. In continuation of our stability studies, we are reporting the isolation and characterization of the major degradation products of the minor steviol glycoside from *S. rebaudiana* namely rebaudioside M under acid conditions at higher temperature.

## 2. Results and Discussion

**Figure 1.**
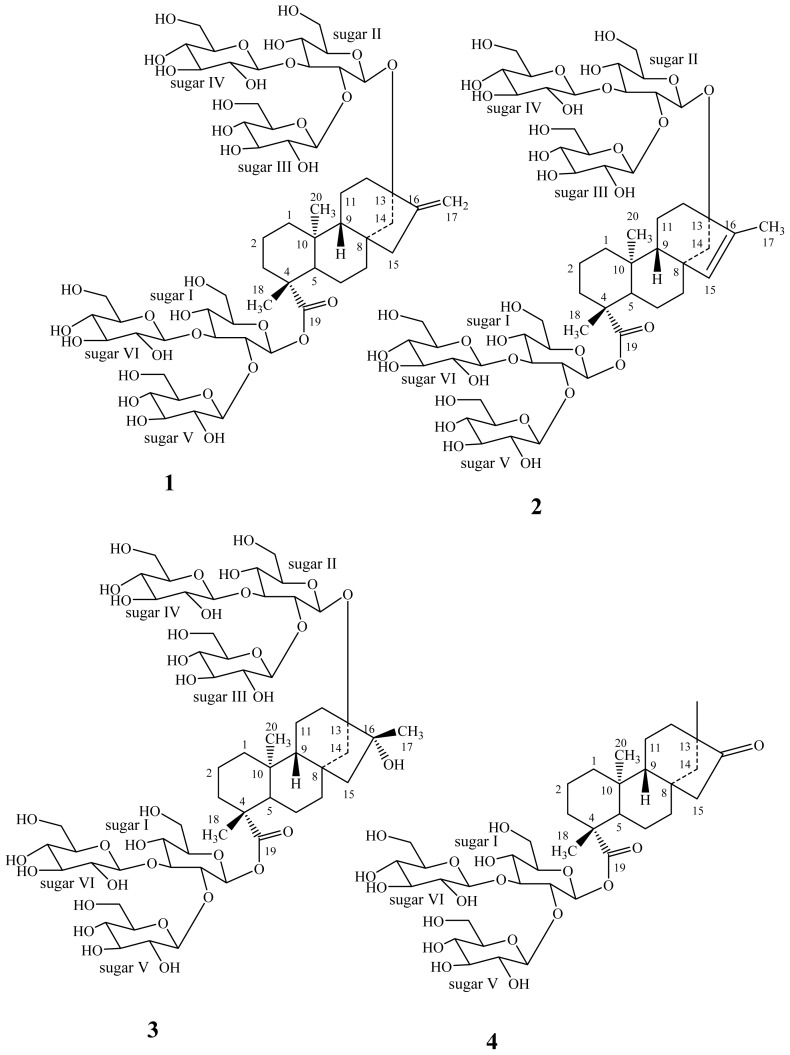
Structures of rebaudioside M (**1**) and its degradation compounds **2**–**4**.

The structures of the degradation compounds **2**–**4** (Figure 1) identified during the course of this study were characterized on the basis of extensive spectroscopic data (^1^H- & ^13^C-NMR, COSY, HSQC, HMBC, MS, MS/MS) and hydrolysis studies.

Compound **2** was obtained as a white powder and its molecular formula was assigned as C_56_H_90_O_33_ from its HRMS, which showed [M + H]^+^ and [M + Na]^+^ ions at *m*/*z* 1291.5439 and 1313.5254, respectively; this was supported by the ^13^C-NMR spectral data. The ^1^H-NMR spectrum of **1** showed the presence of three methyl singlets at δ 1.33, 1.35 and 1.89, eight methylene and two methine protons between δ 0.77–2.69, and a trisubstituted olefinic proton at δ 5.03, suggesting the presence of an *ent*-13-hydroxykaur-15-en-19-oic acid skeleton in its structure [7]. The presence of 15-ene *ent*-kaurane diterpenoid skeleton in **2** was supported by COSY (H-1/H-2; H-2/H-3; H-5/H-6; H-6/H-7; H-9/H-11; H-11/H-12) and HMBC (H-1/C-2, C-10; H-3/C-1, C-2, C-4, C-5, C-18, C-19; H-5/C-4, C-6, C-7, C-9, C-10, C-18, C-19, C-20; H-9/C-8, C-10, C-11, C-12; H-14/C-8, C-9, C-13, C-15, C-16, H-15/C-8, C-14, C-16, C-17, and H-17/C-13, C-15, C-16) correlations. In addition, the ^1^H-NMR spectrum of **1** also showed the presence of six sugar units in its structure by exhibiting the anomeric protons at δ 5.33, 5.44, 5.45, 5.47, 5.81, and 6.33. The presence of six sugars was confirmed as hexoses by the fragment ions corresponding to the successive loss of six hexose moieties from its [M + H]^+^ ion. Acid hydrolysis of **2** afforded d-glucose which was identified by preparing the corresponding thiocarbamoyl-thiazolidine carboxylate derivatives and in comparison of its retention times with the standard sugars as described in the literature [18]. The ^1^H- and ^13^C-NMR values for all the protons and carbons in **2** were assigned on the basis of COSY, HSQC and HMBC correlations and are given in Table 1.

**Figure 2.**
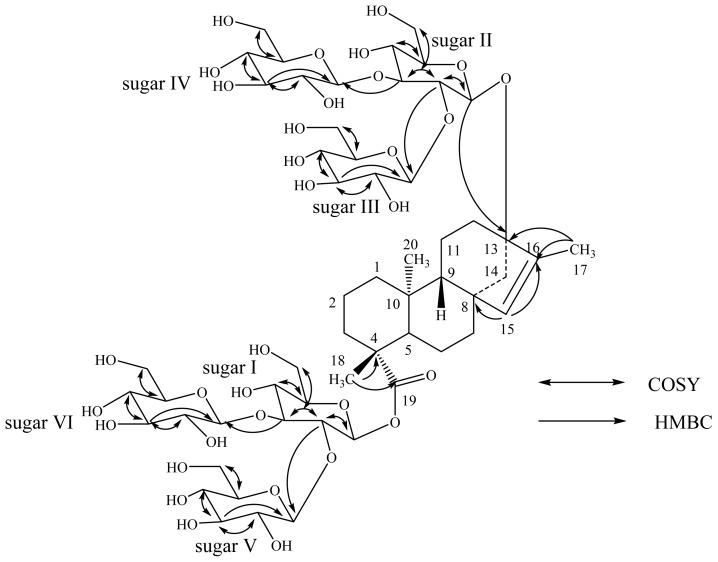
Key COSY and HMBC correlations of **2**.

A close comparison of the ^1^H- and ^13^C-NMR values of **2** with rebaudioside M (**1**) suggested two 2,3-branched β-d-glucotriosyl substituents at C-13 as an ether linkage and as an ester at C-19 positions together with the migration of the exocyclic double bond from C-16/C-17 to C-15/C-16. This was further supported by the ^13^C-NMR values for a trisubstituted double bond between C-15 and C-16 that were observed at δ 134.3 and 144.4, respectively. The large coupling constants observed for the six d-glucose anomeric protons suggested the β-orientation as reported for steviol glycosides. Thus, structure of **2** was established as 13-[(2-*O*-β-d-glucopyranosyl-3-*O*-β-d-glucopyranosyl-β-d-glucopyranosyl) oxy] *ent*-kaur-15-en-19-oic acid 2-*O*-β-d-glucopyranosyl-3-*O*-β-d-glucopyranosyl-β-d-glucopyranosyl ester, which was further confirmed from the key COSY and HMBC correlations as shown in Figure 2.

**Figure 3.**
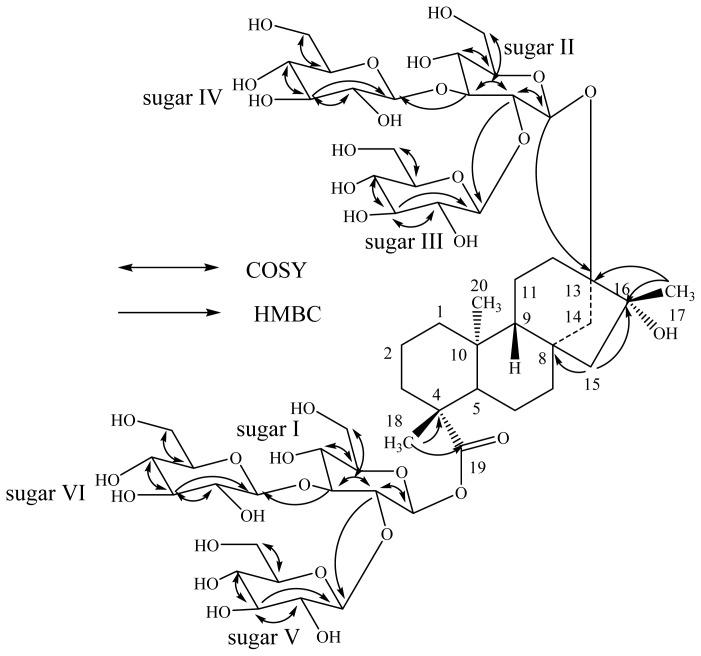
Key COSY and HMBC correlations of **3**.

The molecular formula of compound **3** was determined to be C_56_H_92_O_34_ by the HRMS data that showed (M + H)^+^ and (M + Na)^+^ ions at *m*/*z* 1309.5588 and 1331.5414, respectively. The ^1^H-NMR spectrum of **3** showed the presence of three methyl singlets at δ 1.28, 1.31, 1.32, nine methylene and two methine protons between δ 0.78–2.67. In the absence of any unsaturated protons or carbons together with the appearance of a methyl group at δ 1.32 corresponds to a methyl group connected to a tertiary hydroxyl group suggested the structure of **3** should be similar to *ent*-13,16β-dihydroxykauran-19-oic acid [11]. The presence of tertiary hydroxyl at C-16 position in **3** was supported by the carbon signal appeared at δ_C_ 77.1 in its ^13^C-NMR spectrum. The presence of an *ent*-13,16-dihydroxykaurane skeleton in **3** was further supported by the key HMBC correlations: H-12/C-11, C-13, C-14, C-16; H-14/C-8, C-12, C-13, C-15, C-16; H-17/C-13, C-15, C-16. The ESI MS/MS spectrum of **3** showed the fragment ions corresponding to the successive loss of six hexose moieties from its [M + H]^+^ ion and this was supported by the six anomeric protons observed at δ 5.22, 5.53, 5.63, 5.76, 5.79 and 6.35 in its ^1^H-NMR spectral data. Acid hydrolysis of **3** afforded D-glucose which was identified in comparison of its retention times with the standard sugars as described in **2** [18]. The ^1^H- and ^13^C-NMR values for all the protons and carbons in **3** were assigned on the basis of COSY, HSQC and HMBC correlations (Table 1). A close comparison of the ^1^H- and ^13^C-NMR values of **3** with those of **1** and **2** suggested the presence of six glucose units attached as two 2,3-branched β-d-glucotriosyl substituents at C-13 and C-19 positions of *ent*-13, 16β-dihydroxykauran-19-oic acid, which was supported by the key COSY and HMBC correlations (Figure 3). The large coupling constants observed for the six anomeric protons (Table 2) of the d-glucose moieties suggested their β-orientation. A close comparison of the ^1^H- and ^13^C-NMR values of **3** with 13-[(2-*O*-β-d-glucopyranosyl-3-*O*-β-d-glucopyranosyl-β-d-glucopyranosyl) oxy]-16β-hydroxy-*ent*-kauran-19-oic acid [8] suggested the stereochemistry for the 16-hydroxyl group as β. Thus, the structure was thus deduced to be 13-[(2-*O*-β-d-glucopyranosyl-3-*O-*β-d-glucopyranosyl-β-d-glucopyranosyl)oxy]-16β-hydroxy *ent*-kauran-19-oic acid 2-*O*-β-d-glucopyranosyl- 3-*O*-β-d-glucopyranosyl-β-d-glucopyranosyl ester.

**Figure 4.**
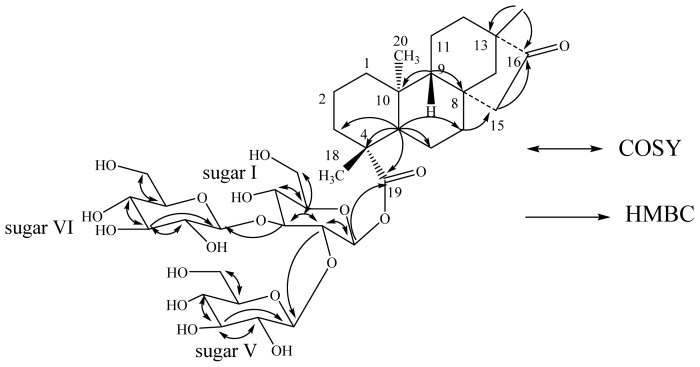
Key COSY and HMBC correlations of **4**.

The molecular formula of compound **4** was deduced as C_38_H_60_O_18_ from the [M + H]^+^ and [M + Na]^+^ ions at *m*/*z* 805.3890 and 827.3707, respectively in the positive ESI mass spectrum. This was also supported by the ^13^C-NMR spectral data. The ^1^H-NMR spectrum of **4** showed the presence of three methyl singlets at δ 0.81, 0.98 and 1.42; nine methylene and two methine protons. Enzymatic hydrolysis of 3 furnished an aglycon that was found to be identical to isosteviol on the basis of its NMR spectral data reported in the literature [19–21]. The presence of the isosteviol skeleton in **4** was supported by the key HMBC correlations: H-12/C-9, C-11, C-13, C-14, C-16, C-17; H-14/C-8, C-12, C-13, C-15, C-16, C-17; H-15/C-8, C-14, C-16; H-17/C-13, C-14, C-16. The ^1^H-NMR spectrum also showed three anomeric proton as doublets at δ 5.35 (*J* = 7.9 Hz), 5.79 (*J* = 7.9 Hz), and 6.19 (*J* = 8.1 Hz), suggesting the presence of three sugar residues in its structure having β-orientation. Acid hydrolysis of **4** afforded d-glucose, confirming the sugar units present in **4** as β-d-glucosyl units similar to **2**, and **3**. The placement of the three β-d-glucosyl moieties in **4** was assigned to be at C-19 as a 2,3-branched β-d-glucotriosyl substituent, based on the key HMBC correlation of the anomeric protons as shown in Figure 4. Based on the results obtained from chemical and spectral studies, **4** was unambiguously assigned as 13-methyl-16-oxo-17-nor-*ent*-kauran-19-oic acid- -*O*-β-d-glucopyranosyl-3-*O*-β-d-glucopyranosyl-β-d-glucopyranosyl ester.

## 3. Experimental Section

### 3.1. Reagents and Chemicals

Ammonium hydroxide (NH_4_OH) was from Fluka (a part of Sigma-Aldrich, Bellefonte, PA, USA), and 85% phosphoric acid (H_3_PO_4_) was from Fisher Scientific (Pittsburgh, PA, USA), all of which were reagent grade. HPLC grade acetonitrile (MeCN) was purchased from Burdick & Jackson (Muskegon, MI, USA). Water was purified using a Millipore system (Billerica, MA, USA).

### 3.2. Plant Material

Leaves of *S. rebaudiana* Bertoni cultivar AKH L1 were obtained from PureCircle (Bandar ENSTEK, Malaysia).

### 3.3. HPLC Conditions

An Agilent (Wilmington, DE, USA) 1200 HPLC equipped with a quaternary pump, a temperature controlled column compartment with additional 6-port switching valve, an auto sampler and a UV absorbance detector, was used for the analysis. A Charged Aerosol Detector (CAD), ESA, Inc. (Chelmsford, MA, USA), was also used for the analysis. The scale on the CAD was 100 pA and the filter was set to medium. The switching valve diverted the first 5.5 min of each injection away from the CAD detector to prevent fouling of the detector. The system was controlled using Waters (Milford, MA, USA) Empower software. The separation HPLC column was maintained at a temperature of 25 °C with a flow rate of 5.0 mL/min. The RP-HPLC employed on a Gemini C_18_ column (250 × 10 mm, 5 μm) (Torrance, CA, USA) with a Gemini Security guard C_18_ cartridge. A binary solvent mobile phase as shown in Table 2 was used for detection and isolation of the three compounds **2**–**4**. The injection volume of each sample was 150 μL, which were kept at ambient temperature while in the auto sampler. In all cases for UV detection, a 4 nm bandwidth was used with a reference wavelength of 210 nm (100 nm band width).

### 3.4. General Instrumentation

NMR spectra were acquired on Bruker Avance DRX 500 MHz instrument (Emory Univeristy, Atlanta, GA, USA) with a 5 mm inverse detection probe using standard pulse sequences. The NMR spectrum was referenced to the residual solvent signal (δ_H_ 8.71, δ_C_ 149.9 for pyridine-*d*_5_), chemical shifts are given in δ (ppm), and coupling constants are reported in Hz. MS and MS/MS data were generated with a mass spectrometer (AMRI, Albany, NY, USA) made by Waters Premier Quadrupole Time-of-Flight (Q-Tof) equipped with an electrospray ionization source operated in the positive-ion mode and Thermo Fisher Discovery OrbiTrap in the positive Positive Mode Electrospray. Samples were diluted with water:acetonitrile (1:1) containing 0.1% formic acid and introduced via infusion using the onboard syringe pump.

### 3.5. Degradation of Rebaudioside M (**1**)

A 0.1 M phosphoric acid solution was made and adjusted to pH 2.0 with concentrated ammonium hydroxide. Ten milligram of **1** was added to 10 mL of phosphoric acid solution. The solution was placed on a heat block at 80 °C for 24 h.

#### 3.5.1. Isolation of Degradation Compounds **2**–**4**

HPLC purification was performed using the method described in Table 2 and the peaks eluting at retention times 7.86, 13.19, and 23.98 min were collected over several injections and dried by rotary evaporation under reduced pressure to yield compounds **2**, **3** and **4** respectively. Samples of the compounds **1**–**4** are available from the authors.

#### 3.5.2. Physical and Spectroscopic Data of Compounds **2**–**4**

13-[(2-*O*-β-d-glucopyranosyl-3-*O*-β-d-gluopyranosyl-β-d-glucopyranosyl)oxy] *ent*-kaur-15-en-19-oic acid (2-*O*-β-d-glucopyranosyl-3-*O*-β-d-gluopyranosyl-β-d-glucopyranosyl) ester (**2**): White powder; ^1^H-NMR (500 MHz, pyridine-*d_5_*, δ ppm) and ^13^C-NMR (125 MHz, pyridine-*d_5_*, δ ppm) spectroscopic data see Table 1; HRMS (M + H)^+^ *m*/*z* 1291.5439 (calcd. for C_56_H_91_O_33_: 1291.5443); 1313.5254 (calcd. for C_56_H_90_O_33_Na: 1291.5262).

13-[(2-*O*-β-d-glucopyranosyl-3-*O*-β-d-gluopyranosyl-β-d-glucopyranosyl)oxy]-17-hydroxy-*ent*-kaur-16β-hydroxy-19-acid (2-*O*-β-d-glucopyranosyl-3-*O*-β-d-gluopyranosyl-β-d-glucopyranosyl) (**3**): White powder; ^1^H-NMR (500 MHz, pyridine-*d_5_*, δ ppm) and ^13^C-NMR (125 MHz, pyridine-*d_5_*, δ ppm) spectroscopic data see Table 1; HRMS (M + H)^+^ *m*/*z* 1309.5588 (calcd. for C_56_H_93_O_34_: 1309.5548); 1331.5404 (calcd. for C_56_H_92_O_34_Na: 1291.5368).

13-Methyl-16-oxo-17-nor-*ent*-kauran-19-oic acid- (2-*O*-β-d-glucopyranosyl-3-*O*-β-d-gluopyranosyl-β-d-glucopyranosyl) ester (**4**): White powder; ^1^H-NMR (500 MHz, pyridine-*d_5_*, δ ppm) and ^13^C-NMR (125 MHz, pyridine-*d_5_*, δ ppm) spectroscopic data see Table 1; HRMS (M + H)^+^ *m*/*z* 805.3890 (calcd. for C_38_H_61_O_18_: 805.3858); 827.3707 (calcd. for C_38_H_60_O_18_Na: 827.3677).

#### 3.5.3. General Procedure for Acid Hydrolysis and Determination of Sugar Configuration in **2**–**4**

Each compound **2**–**4** (500 μg) was hydrolyzed with 0.5 M HCl (0.5 mL) for 1.5 h. After cooling, the mixture was passed through an Amberlite IRA400 column and the eluate was lyophilized. The residue was dissolved in pyridine (0.25 mL) and heated with l-cysteine methyl ester HCl (2.5 mg) at 60 °C for 1.5 h, and then *O*-tolyl isothiocyanate (12.5 μL) was added to the mixture and heated at 60 °C for an additional 1.5 h. The reaction mixture was analyzed by HPLC: column Phenomenex Luna C18, 150 × 4.6 mm (5 u); 25% acetonitrile-0.2% TFA water, 1 mL/min; UV detection at 250 nm. The sugar was identified as d-glucose (*t*R, 12.24 and *t*R, 12.21 min) in all compounds **2**–**4** (*t*R, 12.28 to 12.36 min) [authentic samples, d-glucose (*t*R, 12.38) and l-glucose (*t*R, 11.12 min)] [18].

#### 3.5.4. Enzymatic Hydrolysis of **4**

Compound **4** (250 μg) was dissolved in 2.5 mL of 0.1 M sodium acetate buffer, pH 4.5 and crude pectinase from *Aspergillus niger* (50 μL, Sigma-Aldrich, P2736) was added. The mixture was stirred at 50 °C for 48 h. The product precipitated out during the reaction and was filtered and then crystallized. The resulting product obtained from the hydrolysis was identified as isosteviol, characterized by comparison of its co-TLC with standard compound and ^1^H-NMR spectral data [19–21].

## 4. Conclusions

The degradation of rebaudioside M (**1**) under acidic conditions has been studied at high temperature. The complete ^1^H- and ^13^C-NMR spectral data for the degradation compounds **2**–**4** are reported herewith for the first time based on COSY, HSQC, and HMBC spectroscopic data as well as enzymatic and acid hydrolysis studies.

## Figures and Tables

**Table 1.** ^1^H- and ^13^C-NMR spectral data for compounds **2**–**4** recorded in pyridine-*d_5_* a–c.

Position	2		3		4	
	^1^H	^13^C	^1^H	^13^C	^1^H	^13^C
1	0.77 t (12.8), 1.76 d (12.8)	40.2	0.78 t (13.2), 1.75 d (13.0)	40.3	0.77 td (3.8, 13.2), 1.47 m	39.4
2	1.36 m, 2.25 m	19.3	1.34 m, 2.23 m	19.3	2.02 m	21.6
3	1.03 m, 2.33 m	38.0	1.00 td (3.9, 12.8), 2.32 d (12.3)	38.4	1.07 m, 2.77 d (13.1)	37.3
4	-	44.0	-	43.8	-	44.1
5	1.05 d (12.7)	56.9	1.04 d (12.7)	57.1	1.06 m	57.1
6	2.21 m, 2.31 m	21.8	2.11 d (13.5), 2.43 m	23.1	1.38 m, 2.04 m	19.4
7	1.49 m, 1.89 m	40.0	1.37 m, 1.88 m	42.8	1.34 m, 1.61 m	41.3
8	-		-		-	
9	0.83 d (8.4)	47.0	0.84 d (8.1)	54.8	1.04 m	54.4
10	-	39.6	-		-	
11	1.56 m, 1.67 m	20.9	1.52 m, 1.71 m	19.8	1.04 m, 1.46 m	20.1
12	1.84 t (12.2), 2.36 m	29.9	1.85 m, 2.67 m	31.6	1.24 m, 1.51 m	37.1
13	-	89.6	-	87.6	-	54.2
14	2.17 d (9.8), 2.69 d (9.8)	47.1	2.44 m, 2.58 d (9.9)	40.3	1.83 d (18.5), 2.62 dd (3.1, 18.5)	48.4
15	5.03 s	134.3	1.41 d (14.3), 1.83 m	54.3	1.27 m, 1.36 m	53.8
16	-	144.4	-	77.1	-	220.7
17	1.89 s	12.7	1.32 s	22.2	0.98 s	19.9
18	1.35 s	28.0	1.28 s	27.7	1.42 s	28.8
19	-	176.7	-	176.9	-	175.8
20	1.33 s	16.4	1.31 s	16.0	0.81s	13.8
1′	6.33 d (8.4)	94.5	6.35 d (8.3)	94.5	6.19 d (8.1)	93.0
2′	4.51 t (8.8)	76.6	4.53 t (8.7)	76.4	4.51 t (8.9)	76.8
3′	4.99 m	88.3	4.98 m	88.5	4.27 t m	88.4
4′	4.19 m	69.6	4.21 m	69.7	4.17 m	69.1
5′	4.10 m	78.1	4.14 m	78.3	3.98 m	78.1
6′	4.21 m, 4.33 m	61.5	4.22 m, 4.30 m	61.5	4.24 m, 4.52 m	62.0
1″	5.47 d (8.0)	95.7	5.79 d (6.7)	96.0		
2″	4.13 m	81.1	4.14 m	80.4		
3″	4.89 t (8.9)	87.6	5.09 m	87.9		
4″	4.04 t (8.9)	70.0	4.00 m	69.9		
5″	3.90 m	77.4	4.17 m	77.2		
6″	4.17 m, 4.31 m	62.4	4.13 m, 4.34 m	62.0		
1″′	5.45 d (7.0)	104.5	5.63 d (7.3)	103.8		
2″′	4.15 m	75.6	4.13 m	75.6		
3″′	4.14 m	78.1	4.09 m	78.0		
4″′	4.02 m	72.8	4.08 m	72.2		
5″′	3.77 m	77.3	3.70 m	77.5		
6″′	4.31 m, 4.53 d (10.9)	63.5	4.32 m, 4.49 m	63.2		
1″″	5.44 d (7.5)	103.9	5.53 d (7.9)	103.8		
2″″	3.98 m	75.3	4.02 m	75.3		
3″″	4.46 t (9.0)	77.6	4.55 t (9.0)	77.6		
4″″	4.14 m	71.0	4.22 m	71.0		
5″″	3.99 m	77.8	4.09 m	78.0		
6″″	4.12 m, 4.33 m	61.8	4.13 m, 4.34 m	62.0		
1″″′	5.81 d (6.9)	103.8	5.76 d (7.8)	104.0	5.79 d (7.9)	103.4
2″″′	4.20 m	75.2	4.28 m	74.8	3.99 m	75.7
3″″′	4.20 m	78.2	4.20 m	78.1	4.32 m	78.3
4″″′	4.10 m	73.1	4.13 m	73.5	4.13 m	72.4
5″″′	3.89 m	77.8	3.92	77.6	4.02 m	78.4
6″″′	4.31 m, 4.62 d (11.1)	63.6	4.29 m, 4.64 dd (2.6, 11.6)	63.8	4.36 m, 4.56 m	63.2
1″″′	5.33 d (7.7)	103.9	5.22 d (7.8)	104.0	5.35 d (7.9)	104.5
2″″′	3.97 m	75.1	3.95 m	75.1	4.04 t (8.2)	75.2
3″″′	4.35 t (8.3)	77.7	4.28 m	77.7	4.21 m	78.3
4″″′	4.11 m	70.9	4.08 m	70.8	4.14 m	71.2
5″″′	3.85 m	77.7	3.76 m	77.8	3.85 m	78.7
6″″′	4.12 m, 4.33 m	61.8	4.13 m, 4.34 m	62.0	4.24 m, 4.33 m	61.6

a Assignments made on the basis of COSY, HSQC and HMBC correlations;

b Coupling constants are in Hz;

c Chemical shift values are in δ (ppm).

**Table 2.** RP-HPLC method for the identification and purificationof steviol glycosides **2**–**4**.

Time (min)	% of Mobile Phase A	% of Mobile Phase B
0.0	75	25
20.0	69	31
20.5	50	50
25.0	40	60
25.1	75	25
30.0	75	25

Mobile Phase A: Water; Mobile Phase B: CH_3_CN.
